# Improved Veterinary Service, United States Army

**Published:** 1901-02

**Authors:** 


### SOCIETY PROCEEDINGS.

### IMPROVED VETERINARY SERVICE, UNITED STATES ARMY.

(Continued from page 62.)

The following official rulings show the status referred to:

[CIRCULAR NO. 55.]

HEADQUARTERS OF THE ARMY,
ADJUTANT-GENERAL'S OFFICE,
WASHINGTON, November 23, 1899.

The following decisions have been made and are published to the army for the information of all concerned:

. . . . . . . . . . .

2. STATUS AND ALLOWANCES OF VETERINARIANS. A veterinarian appointed under the Act of Congress approved March 2, 1899, is not a commissioned officer or an enlisted man, but a civil employé. A veterinarian of the second class is entitled to all the allowances or emoluments of a sergeant-major other than his pay proper, which is fixed by law, the same as if he were an enlisted man.—(*Decision Sec. War*, November 7, 1899. —290576 *A. G. O.*)

By command of Major-General Miles.

H. C. CORBIN,
*Adjutant-General.*

RULINGS: PAYMASTER-GENERAL'S OFFICE.

VETERINARIANS: First class, pay $125 per month. Act approved March 2, 1899. G. O. 36, A. G. O., 1899.

*Note:* Under decision of Secretary of War, a veterinarian is a civil employé, and as such is not entitled to service pay, commutation of quarters, mileage, nor to travel pay upon discharge. Par. 2, Cir. 55, A. G. O., 1899, and Dec. 2d, Comp., par. 2087, edition of 1869.

Second class, pay $75 per month. Act approved March 2, 1899. G. O. 36, A. G. O., 1899.

*Note:* Authorized to charge $3.71 per month on monthly pay account, in lieu of clothing allowance. Letter Adjutant-General to Paymaster-General, dated January 27, 1900. Not entitled to increased pay for service, mileage, nor travel pay upon discharge. Same status with regard to civil employé as first class veterinarians.

Senators Sewell and Cockrell, of the Military Committee, took the floor in opposition and were aided by speeches from Senators Lodge, Tillman, Foraker, and Spooner.

Senator Kenney's amendment was adopted by the following vote:

YEAS—25.

| | | |
|---|---|---|
| Allison, | Gallinger, | Nelson, |
| Bacon, | Gear, | Perkins, |
| Baker, | Hale, | Quarles, |
| Chandler, | Hansbrough, | Stewart, |
| Clay, | Kenney, | Taliaferro, |
| Culberson, | McComas, | Teller, |
| Foster, | McCumber, | Turner, |
| Frye, | McEnery, | Wolcott. |
| | Morgan, | |

NAYS—23.

| | | |
|---|---|---|
| Bard, | Kean, | Ross, |
| Bate, | Kyle, | Sewell, |
| Cockrell, | Lodge, | Shoup, |
| Deboe, | Pettigrew, | Spooner, |
| Depew, | Pettus, | Sullivan, |
| Fairbanks, | Platt, Conn., | Vest, |
| Foraker, | Proctor, | Warren. |
| Hawley, | Rawlins, | |

NOT VOTING—39.

| | | |
|---|---|---|
| Aldrich, | Davis, | Martin, |
| Allen, | Elkins, | Mason, |
| Berry, | Hanna, | Money, |
| Beveridge, | Harris, | Penrose, |
| Burrows, | Heitfeld, | Platt, N. Y., |
| Butler, | Hoar, | Pritchard, |
| Caffery, | Jones, Ark., | Scott, |
| Carter, | Jones, Nev., | Simon, |
| Chilton, | Lindsay, | Thurston, |
| Clark, Mont., | McBride, | Tillman, |
| Clark, Wyo., | McLaurin, | Turley, |
| Cullom, | McMillan, | Wellington, |
| Daniel, | Mallory, | Wetmore. |

It then became section 14 of the Army bill, S. 4300.

[13] The Army bill went to the House of Representatives on May 5th, and was taken up for consideration by the Military Committee of the House. Dr. D. E. Salmon, Chief of the Bureau of Animal Industry, and Dr. Huidekoper, of our committee, were heard before the Military Committee. Their testimony in behalf of the needs of a veterinary service appears in the report of the "hearing before the Committee of Military Affairs of the House of Representatives on the bill (S. 4300) entitled An Act to Increase the Efficiency of the Military Establishment of the United States," and in the pamphlet published by our Association in the summer of 1900, entitled "Veterinary Corps, United States Army, Fifty-sixth Congress."

This latter pamphlet included also a distinct argument showing the base of our claims for favorable consideration and an equally distinct statement showing the base of opposition, and calling attention to the fact that the provision for the Veterinary Corps had passed the Senate, opposed by the Military Committee.

[14] During the summer and autumn of 1900 members of the various State Veterinary Associations, and of our Association, furnished the pamphlet just referred to, and other data concerning the need of a Veterinary Corps, to the Honorable Senators and members of the House of Representatives, and, by letter or personal interview, requested their personal consideration of the subject. The large majority of the Senators and of the members of the House of Representatives responded with the statement that they understood the subject, and gave assurance of their support of it.

[15] On June 26, 1900, Dr. Leonard Pearson, then President of our Association and the secretary of our committee, called upon the President, Mr. McKinley, in regard to the bill, and received from him the statement:

PRESIDENT McKINLEY: "I am very much interested in this veterinary matter, and you have my best wishes for success."—June 26, 1900.

[16] They called on the Secretary of War to present additional facts in the case and request his further consideration, but found the Secretary absent. The Adjutant-General, General Corbin, however, was in, and said the Secretary would give a hearing before the next session of Congress.

[17] In November our secretary wrote to General Corbin asking when we could have a hearing, to which we had no reply.

[18] The latter part of November our secretary wrote from New York to the Honorable Mr. Hull asking when he should come to Washington, and suggesting some details in regard to veterinarians for the artillery, and any additional cavalry regiments which might be added.[1]

The following was the reply:

COMMITTEE ON MILITARY AFFAIRS,
HOUSE OF REPRESENTATIVES, U. S.,
WASHINGTON, D. C., November 26, 1900.

COLONEL RUSH S. HUIDEKOPER,
Army and Navy Club, New York, N. Y.

MY DEAR COLONEL: Your favor of the 25th instant received. There will be no necessity of your being here until probably some time next week. I note what you say as to second lieutenants, and will look it up when we take the bill up for consideration. I am,

Very truly yours,
(Signed) J. A. T. HULL.

On December 4th we learned for the first time that section 14 was to be struck out of the bill, S. 4300, and the next afternoon obtained a copy of the bill and found it had been.

[19] We appealed to some friends of the measure, and on December 6th, when the Army bill was before the House, the Hon. General H. H. Bingham, of Pennsylvania, brought the section up, and it was replaced on the bill by a vote of the House.

General Bingham was aided in his advocacy of it by the Hon. Mr. Sulzer, of New York, a member of the Committee of Military Affairs of the House, and by the Hon. Messrs. Adams, Butler, and Green, of Pennsylvania, and Gaines, of Tennessee.

It was opposed in debate by the Hon. Messrs. Hull, Iowa; Mondell, Wyoming; Parker, New Jersey, and Hay, Virginia, of the Committee of Military Affairs, and by Messrs. Steele, of Indiana, and Grosvenor, of Ohio.

The section, replaced without the alteration of a word, became section 20 of S. 4300.

After passing the House the Army bill was returned to the Senate.

Of the original bill,

[1] See 8.

"S. 4300.

AN ACT

[20] To increase the efficiency of the military establishment of the United States. *Be it enacted by the Senate and the House of Representatives of the United States in Congress assembled,*"

There remained the title of the bill, the enacting clause, and the Veterinary Corps section, all of which, without a word of alteration, had passed the Senate on May 4th and the House of Representatives on December 6th.

The other sections which had passed the Senate were struck out and amendments were added by the House.

[21] On reaching the Senate the Army bill was referred to the Military Committee of the Senate.

[22] This Committee struck out the Veterinary Corps section, which had already passed both the Senate and the House of Representatives, and made an amendment as follows:

"SEC. 16. That the grade of veterinarian of the second class in cavalry regiments, United States Army, is hereby abolished, and hereafter the two veterinarians authorized for each cavalry regiment and the one veterinarian authorized for each artillery regiment shall receive the pay and allowances of second lieutentants, mounted. Such number of veterinarians as the Secretary of War may authorize shall be employed to attend animals pertaining to the quartermaster's or other departments not directly connected with the cavalry and artillery regiments, at a compensation not exceeding one hundred dollars per month."

The Military Committee of the Senate reported the bill, with further amendments, on December 20th.

The following testimony was given before the Military Committee of the Senate:

BY THE SECRETARY OF WAR.

[23] Secretary Root: We pass to the Veterinary Corps. You will remember after you reported the bill at the last session the clause providing for a Veterinary Corps, with a colonel at its head, was put in on the floor of the Senate. The same thing has been put in by the House and in the same way. The House committee was opposed to it, as you were opposed to it, and it was put in on the floor of the House. It was put in on statements made, unquestionably in entire good faith, to the effect that there was a general agreement by military authorities in favor of it. The statement was made on the floor of the House that the President was in favor of it. The President[1] and his Secretary of War certainly are not at issue on that question. He is not in favor of it. The statement was made that General Miles was in favor of it.[2] The statement was a mistake; he is not; I have here a written statement to that effect. The statement was made that the Quartermaster-General, General Ludington, who has charge of purchase of horses for the army, was in favor of it. The statement was a mistake; he is not. I have his written authority to that effect.[3] The statement was made that General Merritt was in favor of it.[4] General Merritt made a statement, and it appeared in the *Congressional Record* last session, that he is in favor of a Veterinary Corps, but totally opposed to the creation of a new bureau in the War Department, and that is what this

[1] The President. See 2, 3, and 15.

[2] General Miles. See 8.

[3] See 6.

[4] From letter of General Wesley Merritt.

creates. The provision is for a colonel, four majors, ten captains,[1] ten first lieutenants, and twenty second lieutenants. The colonel is to report directly to the Secretary of war. Now, take the full force and the strength of that. What does it mean? Where is the office of this colonel to be? He is to report to the Secretary of War. That means he is to be in the War Department. And you have got another bureau of the War Department. The officers of the corps will be under the direction of a colonel, just as the officers of the Engineer Corps are under the direction of the Chief of Engineers.

The colonel of cavalry, who, when he is ordered out, ought to be held responsible for having not only his men but his horses fit for duty, is relieved from that responsibility because the Secretary of War is charged with it through his new bureau of the War Department—through this chief of the Veterinary Corps. And I protest against it as being the reversal of all the principles of sound administration and an increase of the evil that now oppresses the American army, which is an excess of multiple command—too many bureaus and too much staff control. I do not object to having competent veterinarians—I do not object to their having rank which will enable them to take places in the headquarters and be treated on a different basis from the enlisted man;[2] but I submit that the officer who has charge of the horses of a regiment should be under the command of the colonel of the regiment, and not an independent officer receiving constant instructions and communications from the Secretary of War through the head of the bureau, which will be superior to the control and the directions and the discipline of the colonel of the regiment.[3]

Senator Cockrell: Suppose the colonel of cavalry was ordered to make a certain movement, to take with him a certain number of men.[4] His veterinarian examines the horses, we will say, and finds a certain number unfit for duty, and his commands prohibit those horses from being used. What would the colonel do?

Secretary Root: I do not know; he could not do anything.

Senator Proctor (addressing Senator Cockrell): You would shut the veterinarian up in the guard-house; that is what you would do.

Senator Cockrell: Suppose the veterinarian says the horses are unfit to be ridden, and the colonel then orders them out anyway and the veterinarian stops them; there would be a little collision. We all know what would be the result if there was a good colonel in command. But suppose those men are commissioned and the veterinarian in a regiment has equal authority with the colonel of cavalry, then it would be a difficult matter to settle.

Secretary Root: I do not know any principle so important in administration as that responsibility shall be fixed. It does not make much difference how you change leaders if you can fix the responsibility on somebody—hold somebody responsible. You will then get along. But just as soon as you begin to divide up responsibility, so that you shift it off on to somebody else, you are gone.

### By the Adjutant-General.

General Corbin: I object particularly to the introduction of the Veterinary Corps. We should not object to the veterinarians being commissioned,

1 "I am heartily in favor of the organization of a Veterinary Corps for the United States Army. The need of a properly organized corps has long been recognized, especially by officers whose service has been with mounted troops. The cost of such a corps would be small compared with the increase in the efficiency of the animals of the mounted and transportation services and the value of what it would save to the government. To make such a corps properly effective the veterinarians composing it should, of course, have commissioned rank."

2 See 7, Amendment. 3 See 3.

4 Imagine such a military hypothesis!

but we do not want an additional staff corps. There is no need of it. We have too many staff corps now. The policy to increase the number of staff corps is bad.

Senator Sewell: We made the veterinarian a second lieutenant a year or two ago.

General Corbin: Yes; but it did not become a law.

Senator Burrows: But they get the pay of second lieutenants.

General Corbin: Yes.

Senator Burrows: I want to understand this. Page 24 of the bill, as it passed the House, section 24, relates to the Veterinary Corps and runs from the bottom of that page to the bottom of page 25. What criticism have you of that? Please point out the lines.

General Corbin: In short, I would eliminate the whole proposition to make any additional staff corps, call it what name you please. There is no necessity for such action. The Secretary of War has looked into the matter. Every cavalry regiment has an efficient veterinary surgeon now, with pay of second lieutenant, and the colonel of the regiment should be the man who would conduct the examination and conduct all that relates to the health of his troop, and the captain of his troop the same way. We do not need an officer in Washington to do that.

Senator Sewell: The captain of the troop arrives at that position after fifteen or twenty-five years of service, and he is the most accomplished veterinarian there is.

General Corbin: Yes, exactly; and the same way with officers in the Quartermaster's Department.

Senator Cockrell: Are the veterinarians sufficiently provided for in the bill as introduced?

General Corbin: Yes; and that is entirely satisfactory. *The present service is efficient and economical, satisfactory to everybody*, and the only people who desire a change are the men who want to get in as colonels and lieutenant-colonels and majors.

Senator Cockrell: Then you would strike out this section entirely?

General Corbin: Yes.

The Chairman: All you recommend, then, is to strike out section 20?

General Corbin: Yes, sir. The Secretary of War has examined the matter carefully and he is thoroughly convinced about it. I think 99 per cent. of the officers of the army would sustain what I am saying here. I doubt if you can get a cavalry officer in the entire service to say that he wants to be directed how to treat his horses from a bureau in Washington.[1]

In the debate in the Senate on January 7th Senator Kenney made this criticism of the "Report of the Senate Committee:"

I desire to call the attention of the Senate to the report of the Committee on Military Affairs covering that proposition and to invite their attention to the statement—no doubt not intentionally made by the Committee, but nevertheless it is misleading—which I read from the report:

"An amendment not reported by the Committee—"

This follows the report of the Committee dealing with the action of the House on the Senate bill, leaving the impression upon the mind of any Senator who might examine this report that it was the action of the House and not of the Senate. The report reads as follows:

[24] "An amendment not reported by the Committee last session was inserted in Senate bill 4300, providing for a formidable Veterinary Corps."

Mr. President, the Committee says the provision was inserted in the House, when, in point of fact, it was inserted in the Senate on my motion to agree to the amendment establishing that corps.

[1] Nineteen out of twenty army officers will state the contrary.

The report continues:

"The Senate Committee proposes to strike out the section providing for such a corps, which was inserted in the House, and insert in lieu therefor section 16, which in the judgment of cavalry officers and quartermasters grants to the army a sufficient number of veterinarians, with sufficient rank and pay."

That it is not a formidable corps, see a further statement of Senator Kenney's:

"In the English army, Mr. President, the proportion of veterinarians to the number of troops is 136 to 17,586; in this country, under the provisions of this amendment, the proportion would be 36 to 34,000; in Austria-Hungary there are 180 veterinarians to 47,380 troops; in Belgium, 35 veterinarians to 6064; in France, 603 veterinarians to 80,000; in Prussia, 501 to 53,270; in Spain, 236 veterinarians to 14,376. So in the scheme now under consideration the proportion of veterinary surgeons to the troops will be less than that of any of the well-regulated armies of the European nations." (For troops, read horses.)

Every veterinarian in these armies is a commissioned officer.

[25] On the morning of this Senate debate the newspapers of the country contained a long letter of transmittal from the Secretary of War to Hon. Joseph R. Hawley, Chairman Committee on Military Affairs, United States Senate, with the following:

New York State Veterinary Medical Society,
Binghamton, N. Y., December 31, 1900.

Hon. Elihu Root,
Secretary of War, Washington, D.C.

My Dear Sir: The position you take in relation to the Veterinary Corps bill is quite in accord with the sentiments of many of the best veterinary practitioners in the country.

The profession at large is not at heart asking for the enactment of this measure. It is not clear to the minds of many of the necessity for a veterinary bureau, while we all agree as recognizing the importance of a high-grade service for the cavalry. The profession all over the country has been urged by the gentlemen who are fathering the scheme to render every possible aid in the endeavor to obtain a favorable consideration by Congress, and no doubt much of the request in behalf of the bill from constituents to Senators and Representatives is in obedience to this solicitation.

Our Society at its annual meeting, September last, did not consider this matter, although Dr. Huidekoper was present, and it is presumed he was there for the purpose of enlisting support; but learning there was some opposition, no doubt considered that no indorsement from the Society was safer than an average resolution.

I am not speaking for the Society as against the bill, but simply to say that the State Society is not on record in behalf of the bill. As a private practitioner I have written our Senator, Hon. Chauncey M. Depew, stating my objections to this measure.

With sincere consideration and esteem, I beg to be,

Very respectfully,
Claude D. Morris, V.S.,
Secretary.

A copy of the Secretary of War's letter of transmittal and of Claude D. Morris's letter were presented to the Senate by Senator Sewell later in the day.

At the opening of the Senate, Senator Hawley asked permission to print additional copies.

Mr. Hawley: I ask for permission to present out of order a resolution to print additional copies, which we shall probably need this afternoon.

The President *pro tempore:* It will be received and lie on the table for the present.

Mr. Hawley: I should like to have an exception made and to have the order entered now, because it relates to the Veterinary Corps, which may be the first thing to be considered to-day on the Army bill.

The President *pro tempore:* Is there objection to the reception of the resolution now? The Chair hears none.

The order was read and agreed to as follows:

*Ordered,* That 500 copies of a letter from the Secretary of War submitting letters concerning the proposed Veterinary Corps be printed for the use of the Senate.—*Congressional Record,* January 7th.

In an hour these copies were on the desks of the Senate.

Later in the afternoon Senator Gallinger said:

The Senator from New Jersey laid great stress upon a letter from the Secretary of War—a letter that was laid upon my desk as I entered the chamber; a letter that seems to have been ordered printed this very day, and undoubtedly it was marked "Rush," and it is back here to influence our votes on this bill.

Now, Mr. President, the opinion of the Secretary of War is well known, and it is barely possible that in the debate last May the opinions of some of the high army officers may have been misstated. I do not know how that may be. I do know that army officers change their minds. I do know that the Adjutant-General of the Army not long ago wrote a letter in opposition to the army canteen and that he has now given testimony in favor of it, and it is just possible that some of these other army officers have changed their minds in reference to this veterinary provision.

But what the Senator from New Jersey seemed to lay great stress upon was a letter from Dr. Claude D. Morris, a veterinarian living in Binghamton, N. Y., who seems to be the Secretary of the New York State Veterinary Medical Society.

Now, Mr. President, if I am correctly informed, this Secretary might be called an "accidental" secretary, and the representations he makes here are not the views of the Society of which he is Secretary. As I understand the matter, the New York Veterinary Medical Society has given its sanction to this bill by vote, and it was not necessary for that Society to pass another series of resolutions indorsing it, as Dr. Morris seems to think ought to have been done. I say, Mr. President, that Claude D. Morris does not represent the New York State Veterinary Medical Society on this subject. As I entered the Senate chamber I was handed some telegrams. I do not know why they were sent to me, but they were sent to me, and I want to read two telegrams bearing on this very point. The first is dated Brooklyn, N. Y., January 7, 1901.

BROOKLYN, N. Y., January 7, 1901.

SENATOR GALLINGER,

United States Senate, Washington, D. C.:

Letter of C. D. Morris, Secretary New York State Veterinary Medical Society, to Secretary Root, published in morning papers,[1] stating Society

[1] See 25.

not in accord with amendment to Army bill creating Veterinary Corps, is without authority and absolutely false. Profession of State almost to a man has worked night and day for it, and will be struck a staggering blow if it does not pass.

ROSCOE R. BELL,
President New York State Veterinary Medical Society,
Editor "American Veterinary Review."

The next telegram reads as follows:

ALBANY, N, Y., January 7, 1901.

SENATOR GALLINGER,
United States Senate, Washington, D. C.:

The New York State Veterinary Medical Society is in favor of a Veterinary Corps in the Army Reorganization bill. Secretary C. D. Morris individually is against it, but not the Society.

WM. H. KELLY,
Chairman Legislative Committee New York State Veterinary Medical Society
and State Secretary American Veterinary Medical Association.

Now, Mr. President, if I had been here and these telegrams had been on my desk when this document was ordered printed I would have asked that those telegrams might have been printed in conjunction with it, because it shows that while this one man has made haste to write a letter to the Secretary of War, which the Senate has made haste to print, to show that the New York State Veterinary Medical Society is opposed to this bill, the President of that Society and the Chairman of the Legislative Committee of that Society, speaking for the Society with more authority than a Secretary could possibly speak, say that his statements are false and that the Society is in favor of this measure.

I have three other telegrams which I desire to place in the *Record*. They are as follows:

WEST PHILADELPHIA, PA., January 7, 1901.

HON. JACOB H. GALLINGER,
Washington, D. C.:

All veterinarians in Pennsylvania and good veterinarians everywhere are in favor of the Army Veterinary bill.

S. J. J. HARGER,
President State Veterinary Society, University of Pennsylvania.

WEST PHILADELPHIA, PA., January 7, 1901.

HON. JACOB H. GALLINGER,
Washington, D. C.:

The veterinary organizations and the schools of the country all desire Army Veterinary bill to become law.

W. HORACE HOSKINS,
Ex-President American Veterinary Association.

WEST PHILADELPHIA, PA., January 7, 1901.

HON. JACOB H. GALLINGER,
Washington, D. C.:

The veterinary profession, with 8000 members, is solidly for the Army Veterinary bill. Sincerely hope, for good of army, it will pass to-day.

LEONARD PEARSON,
Pennsylvania State Veterinarian.

Now, Mr. President, I read these telegrams to show that this document that has been placed upon our desks and upon which so much stress has been laid is of no earthly consequence whatever. Claude D. Morris, Secretary of the New York State Veterinary Medical Society, seems to speak for himself and for nobody else.

The Senator from Massachusetts [Mr. Lodge], when discussing this amendment, said that everybody is in favor of giving the veterinarians suitable rank and suitable pay. The Senator ought to have made an exception, and that exception ought to have been the Committee on Military Affairs of the Senate of the United States, which has reported an amendment which does not give them any rank whatever.

[26] It is true that during this debate since I entered the Senate chamber some members of that Committee have said that they are willing to yield this question of rank and give veterinarians rank of some kind. That reminds me of what a distinguished judge in my State once said. He was Chief Justice of the Supreme Court, and alluding to a certain matter he said it was "late, reluctant, and unimportant." That is precisely the situation that the Committee on Military Affairs finds itself in to-day in reference to this matter. They have taken the ground that these men should not have rank. They have insisted upon that in season and out of season, and now they say that they are willing to give rank, doubtless provided the rank suits the Committee. We fought that battle here last May, and we succeeded in putting a provision in the bill which gave these men rank. Now they say to-day, Why, we are in favor of giving them rank of some kind ; but notwithstanding that fact—

The debate on the subject is in the *Congressional Record* of January 7, 1901.

It was a foregone conclusion that the vote had been decided before the debate.

[27] The Senate amendment, leaving the veterinarian a mere civil employé, without rank, as under the act of March 2, 1899, passed by the following vote:

YEAS—43

| | | |
|---|---|---|
| Aldrich, | Frye, | Pettigrew, |
| Allison, | Hanna, | Pettus, |
| Bard, | Hansbrough, | Platt, Conn., |
| Bate, | Harris, | Platt, N. Y., |
| Berry, | Hawley, | Proctor, |
| Burrows, | Hoar, | Sewell, |
| Butler, | Lindsay, | Spooner, |
| Caffrey, | Lodge, | Stewart, |
| Carter, | McComas, | Teller, |
| Cockrell, | McCumber, | Thurston, |
| Daniel, | McMillan, | Turley, |
| Deboe, | Mallory, | Vest, |
| Dolliver, | Morgan, | Warren. |
| Fairbanks, | Nelson, | |
| Foraker, | Perkins, | |

NAYS—5.

| | | |
|---|---|---|
| Clay, | Hale, | Kenney. |
| Gallinger, | Heitfeld, | |

NOT VOTING—38.

| | | |
|---|---|---|
| Allen, | Jones, Ark., | Rawlins, |
| Bacon, | Jones, Nev., | Scott, |
| Baker, | Kean, | Shoup, |
| Beveridge, | Kyle, | Simon, |
| Chandler, | McBride, | Sullivan, |
| Chilton, | McEnery, | Taliaferro, |
| Clark, | McLaurin, | Tillman, |
| Culberson, | Martin, | Towne, |
| Cullom, | Mason, | Turner, |
| Depew, | Money, | Wellington, |
| Dillingham, | Penrose, | Wetmore, |
| Elkins, | Pritchard, | Wolcott. |
| Foster, | Quarles, | |

(To be continued.)

